# Aniridie et aphaquie post traumatique

**DOI:** 10.11604/pamj.2014.18.24.4012

**Published:** 2014-05-07

**Authors:** Othmane Belhadj, Zouheir Hafidi

**Affiliations:** 1 Service d'Ophtalmologie A, Hôpital des spécialités, Université Mohammed V, CHU Rabat, Maroc

**Keywords:** Aniridie, aphaquie, traumatisme oculaire, aniridia, aphakia, eye trauma

## Image en medicine

Il s'agit d'un patient âgé de 30 ans sans antécédents notables, victime d'un traumatisme contusif sévère au niveau de son oeil droit suite à une agression, ce qui a occasionné une baisse d'acuité visuelle brutale. Devant la non amélioration de cette symptomatologie, le patient s'est présenté 5 jours plutard aux urgences. L'examen à l'admission à mis en évidence au niveau de l'oeil traumatisé, une acuité visuelle réduite à une perception lumineuse. A la lampe à fente nous avons noté une absence totale de l'iris, une aphaquie (absence de cristallin), du vitré en chambre antérieure avec un fond d'oeil inaccessible à cause d'une hémorragie intravitréenne dense. L’échographie a objectivé une luxation complète du cristallin au niveau de la cavité vitréenne sans décollement de rétine associé. Le patient à bénéficié d'une vitrectomie postérieure avec équipement de l'aphaquie par une lentille de contact. En post opératoire le patient à présenté une photophobie invalidante, ce qui a motivé un équipement par un implant à fixation sclérale avec collerette à iris artificiel réduisant ainsi la gêne fonctionnelle. Les traumatismes oculaires associés aux atteintes irido cristalliniennes sont fréquents, cependant les aniridies totales sont relativement rares et posent un problème de prise en charge. En effet la gestion des aniridies dépend du tissu irien résiduel. En cas d'aniridie partielle une pupilloplastie peut etre envisagé et l'iris peut constituer un plan d'implantation. En cas d'aniridie totale des diaphragmes peuvent palier au problème de photophobie à condition d'avoir un plan capsulaire respecté. Un lit capsulaire défectueux rend cet équipement plus difficile. Dans ce cas on peut envisager un implant à fixation sclérale avec collerette à iris artificiel.

**Figure 1:**
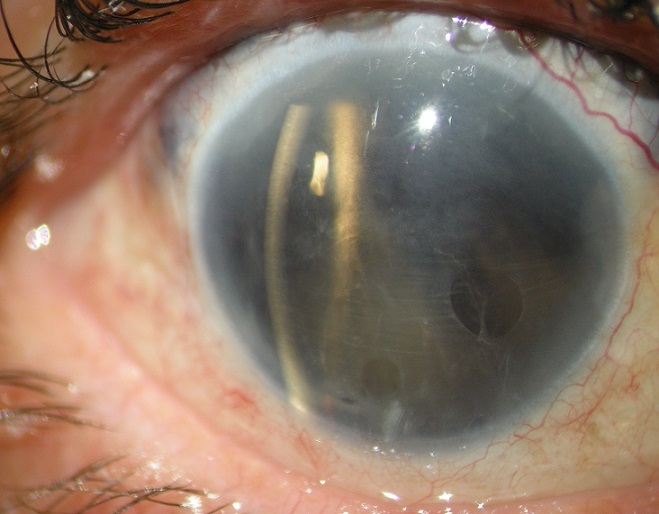
Aspect à la lampe à fente mettant en évidence une aniridie complète avec une aphaquie et du vitré en chambre antérieure

